# Do the current cases reported to the WHO provide a realistic incidence rate of countries infected with COVID-19?

**DOI:** 10.1080/20008686.2020.1751917

**Published:** 2020-04-10

**Authors:** Ghobad Moradi, Amjad Mohamadi Bolbanabad, Bakhtiar Piroozi, Ehsan Mostafavi, Arshad Veysi, Azad Shokri

**Affiliations:** a Social Determinants of Health Research Center, Research Institute for Health Development, Kurdistan University of Medical Sciences, Sanandaj, Iran; b Department of Epidemiology and Biostatistics, Research Centre for Emerging and Reemerging Infectious Diseases, Pasteur Institute of Iran, Tehran, Iran; c Zoonoses Research Center, Research Institute for Health Development, Kurdistan University of Medical Sciences, Sanandaj, Iran

**Keywords:** COVID-19, emerging infectious disease, infectious disease reporting, international health regulations

In compliance with the International Health Regulations (IHR), endorsed by 196 countries in 2005, it has been agreed that all World Health Organization (WHO) member states will contribute to prevent, protect against, control, and provide an urgent response to the international spread of disease, avoid unnecessary interference with international traffic and trade, ensuring global health security and detect, evaluate and timely report public health events and threats [1]. The WHO plays a coordinating role to help countries to build their capacities of detection, assessment and report of public health events [2]. IHR implementation by committed countries may face serious challenges, including failure to detect and control outbreaks at an early stage due to lack of logistical infrastructure and equipment, and fear of the effects of the rapid closure of international borders that might have detrimental impacts on the tourism industry as well as the country’s economy [3–6].

Emerging and re-emerging infectious diseases have been a global health threat in recent years. Since the global outbreak of HIV/AIDS that began in the early 1980s, many new microorganisms have been identified, many of which have spread worldwide, including severe acute respiratory syndrome (SARS), Middle East respiratory syndrome (MERS), Ebola, Zika, and avian influenza. Given the international flights and other international transportation routes taking place daily in and between all countries around the world, any communicable disease has the potential to spread worldwide very fast [7]. Therefore, more attention must be paid to communicable diseases as a matter of global security and the threat to global health.

A novel coronavirus named SARS-CoV-2 that causes the condition COVID-19 emerged from Wuhan, China in late December 2019. As of 8 March 2020, more than 100,000 cases of COVID-19 in 103 countries had been confirmed across the world. After China, Italy, Iran, and South Korea had the highest number of reported cases [8].

**Figure 1.**
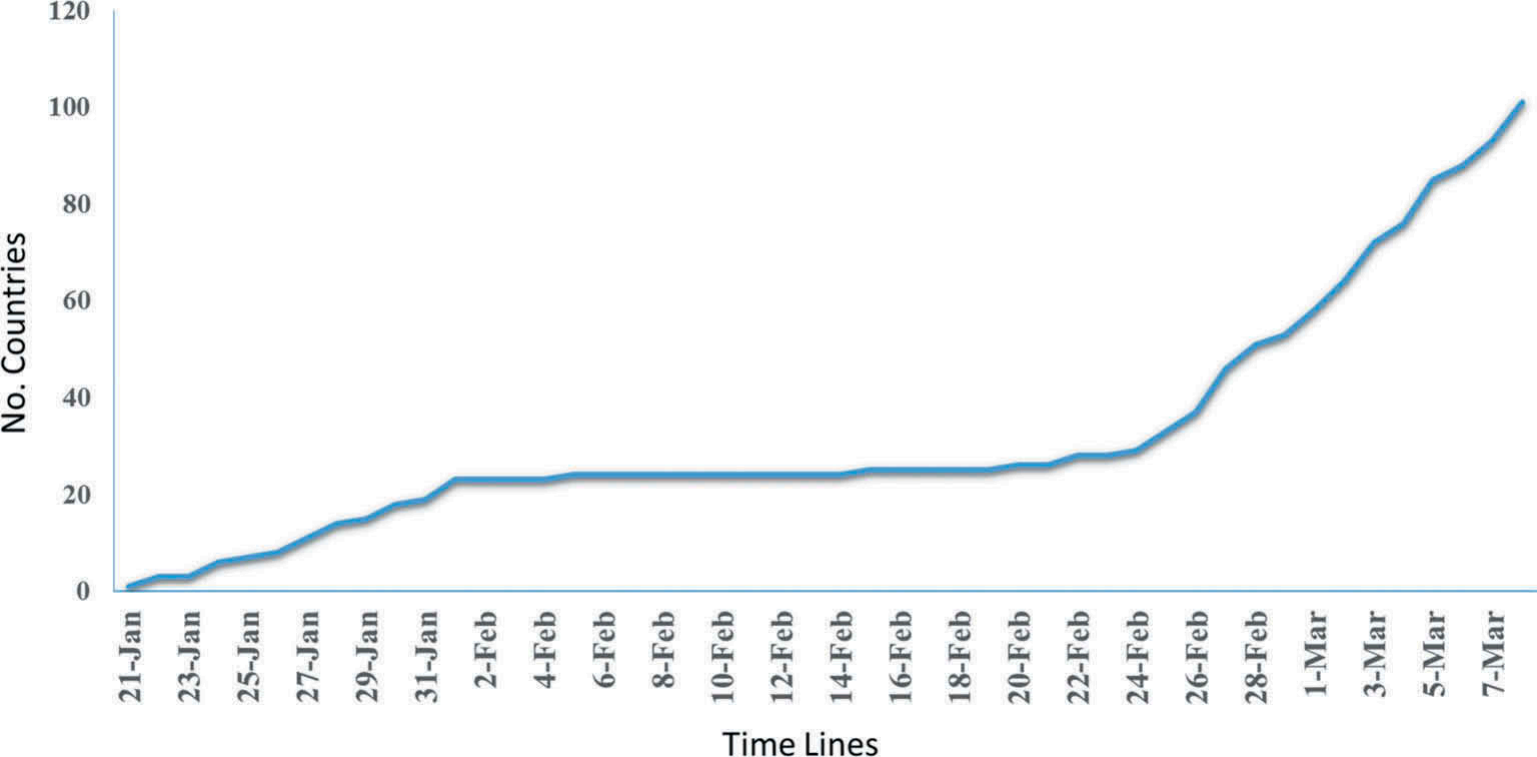
Trend of COVID-19 reporting countries from February 21 to 7 March 2020

Figure 1 demonstrates that during a long period of time, few countries in the world have reported COVID-19 cases, although, from 24 February onwards, countries’ reported cases started to increase rapidly. Considering the origin of the disease, China, having around one-sixth of the world’s population and a relationship with a wide range of countries across the world, this question arises how true are these non-reporting cases of many countries over a long period of time from 31 January to 24 February. Additionally, considering the high transmissibility characteristic of the virus [9] and asymptomatic forms of the disease made it possible to expect disease outbreaks in more countries. Stigma from states’ reporting and fear of economic and social consequences seem to be a deterrent for some countries in reporting positive cases. The accelerated reporting by countries at one point may be due to diminished fear of consequences because of disease reporting in other countries.

**Figure 2.**
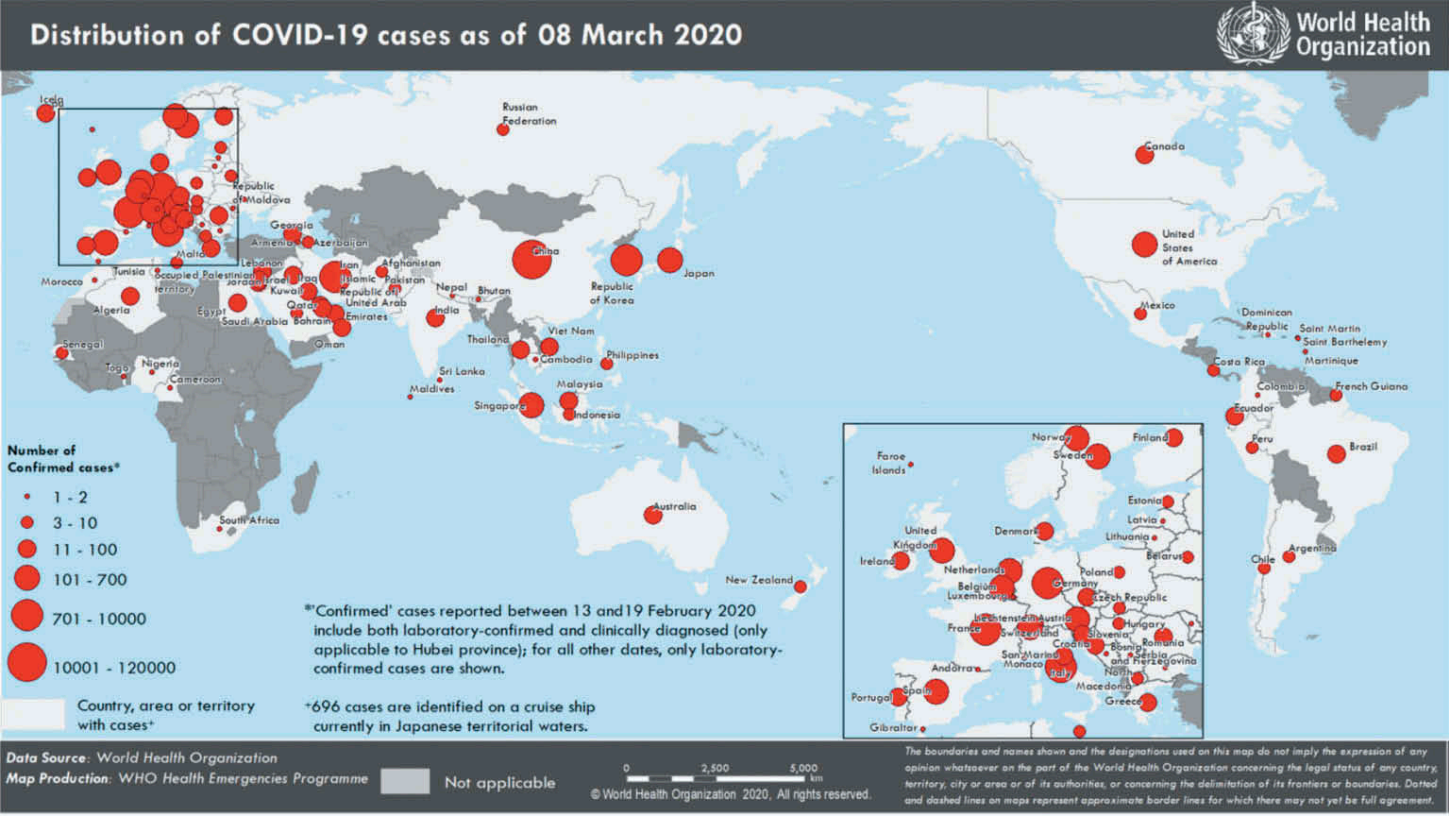
Distribution of COVID-19 reporting countries by 7 March 2020 [8]

A glance at the country distribution map shows that some countries have reported no or limited cases of COVID-19 despite the majority of the surrounding countries' reporting cases, which needs to be justified given the high transmissibility of the disease (Figure 2). Failure to report by some countries seems to be due to poor infrastructure in detecting cases [5]. In such cases, the WHO should be more proactive in providing support.

It seems the current WHO approach regarding reporting cases of emerging diseases and pandemics is not adequate. For contagious diseases that may have high pathogenicity and transmissibility, delays in reporting new cases can have irreparable catastrophic effects across the globe. It is recommended that the IHR rules regarding emerging diseases reporting be revised. Approaches such as those are employed to control doping at the Olympics or athletic federations can be helpful. Unannounced visits and sample screening for targeted diseases can be a good way to diagnose such a disease. Adopting such approaches can improve global health at the time of dangerous emerging diseases pandemics.
